# Rational protein engineering of a ketoacids decarboxylase for efficient production of 1,2,4-butanetriol from arabinose

**DOI:** 10.1186/s13068-023-02414-z

**Published:** 2023-11-13

**Authors:** Xiaolin Shen, Hongchao Xu, Tong Wang, Ruihua Zhang, Xinxiao Sun, Qipeng Yuan, Jia Wang

**Affiliations:** 1 State Key Laboratory of Chemical Resource Engineering, Beijing University of Chemical Technology, 15 Beisanhuan East Road, Chaoyang District, Beijing, 100029 China; 2 College of Engineering, The University of Georgia, Athens, GA 30602 USA

**Keywords:** Metabolic engineering, Synthetic biology, *Escherichia coli*, 1,2,4-butanetriol, Arabinose

## Abstract

**Background:**

Lignocellulose, the most abundant non-edible feedstock on Earth, holds substantial potential for eco-friendly chemicals, fuels, and pharmaceuticals production. Glucose, xylose, and arabinose are primary components in lignocellulose, and their efficient conversion into high-value products is vital for economic viability. While glucose and xylose have been explored for such purpose, arabinose has been relatively overlooked.

**Results:**

This study demonstrates a microbial platform for producing 1,2,4-butanetriol (BTO) from arabinose, a versatile compound with diverse applications in military, polymer, rubber and pharmaceutical industries. The screening of the key pathway enzyme, keto acids decarboxylase, facilitated the production of 276.7 mg/L of BTO from arabinose in *Escherichia coli*. Through protein engineering of the rate-limiting enzyme KivD, which involved reducing the size of the binding pocket to accommodate a smaller substrate, its activity improved threefold, resulting in an increase in the BTO titer to 475.1 mg/L. Additionally, modular optimization was employed to adjust the expression levels of pathway genes, further enhancing BTO production to 705.1 mg/L.

**Conclusion:**

The present study showcases a promising microbial platform for sustainable BTO production from arabinose. These works widen the spectrum of potential lignocellulosic products and lays the foundation for comprehensive utilization of lignocellulosic components.

## Background

Lignocellulose stands as the most abundant non-edible feedstock available on earth, with an annual production of 2 × 10^11^ metric tons [1]. Using this cost-effective and renewable raw material in biological processes to produce chemicals, fuels and pharmaceuticals holds significant promise for mitigating greenhouse gas emission, reducing dependence on petroleum and decreasing environmental pollution [2]. Glucose, xylose and arabinose are the three most abundant components in lignocellulose [3]. Efficiently converting these sugars into high-value chemicals is essential for ensuring the economic viability of lignocellulosic fermentation [4]. In recent years, glucose has been the most frequently employed carbon source for building microbial production platforms [5]. Additionally, significant progress has been made in the bioconversion of xylose into desired products, such as 1,4-butanediol [6], muconic acid [7] and 3,4-dihydroxybutyric acid [8]. Despite arabinose being the second most abundant pentose in lignocellulosic biomass after xylose, it has received less attention for producing value-added products. Thus, it is of great importance to establish innovative microbial platforms that can metabolize arabinose to expand the repertoire of products, ensuring the comprehensive and efficient utilization of lignocellulose.

1,2,4-Butanetriol (BTO) is a versatile non-natural compound that has been extensively used in military, polymer, rubber and pharmaceutical industries [9, 10]. It serves as the direct precursor for the production of butanetriol trinitrate, a renowned propellant and energetic plasticizer that has the potential to replace nitroglycerin [11]. It also can be used to synthesize polyurethane foams, offering similar compression and bending properties as natural rubber [12]. Additionally, BTO plays a crucial role as a recording agent in the formulation of high-quality inks that possess both anti-feathering and penetrability properties [13]. Furthermore, BTO acts as a building-block for the synthesis of cholesterol-lowering drugs like Zetia and Cresto, as well as the anti-HIV drug Amprenavir [14, 15].

Currently, the industrial production of 1,2,4-butanetriol (BTO) predominantly relies on chemical synthesis methods. These methods include the hydrogenation of malic acid using NaBH_4_ as a catalyst [16] and the hydroformylation of glycidol followed by the reduction of the resulting products catalyzed by transition metal carbonyl compounds [17]. Both of these approaches require high-temperature and high-pressure conditions, employ costly catalysts, and result in the formation of various by-products, all of which contribute to low product yields. Alternatively, the establishment of microbial cell factories for BTO synthesis presents an environmentally friendly and sustainable approach.

Up to now, both glucose and xylose have been employed for BTO production. For instance, BTO can be synthesized from glucose using malate as an intermediate through six sequential enzymatic bioreactions catalyzed by malate thiokinase, succinate-semialdehyde dehydrogenase, 4-hydroxybutyrate dehydrogenase, 4-hydroxybutyrate CoA-ligase and alcohol dehydrogenase. The screening of pathway enzymes has enabled the production of BTO using glucose as the sole carbon source in *E. coli* [18]. The highest BTO titer was achieved using xylose as the carbon source. An international patent disclosed a method for converting xylose into BTO via four sequential enzymatic bioreactions catalyzed by xylose dehydrogenase, xylonate dehydratase, 2-keto acid decarboxylase and alcohol dehydrogenase. The most efficient engineered *E. coli* strain generated 18 g/L BTO with a yield of 0.55 mol/mol xylose [19]. More recently, this BTO biosynthetic pathway was introduced into *Saccharomyces cerevisiae*. By improving the NADH/NADPH ratio and supply, it enabled the production of 6.6 g/L BTO xylose with a molar yield of 0.57 [20]. Despite recent advancements in advancing bio-based BTO production, there are relatively few reports on the synthesis of BTO using arabinose.

In this work, we present the design and construction of an artificial biosynthetic pathway to produce BTO from arabinose in *E. coli*. Screening of the pathway key enzyme 2-keto-3-deoxy-arabonate decarboxylase enabled the successful assemble of the entire pathway. To further improve its catalytic efficiency, protein engineering was employed for identifying and screening candidate mutants with enhanced activity. Subsequent in vitro enzyme assays were performed to characterize the kinetic parameters of these mutants. The results showed that the engineered decarboxylase displayed nearly threefold higher catalytic activity toward 2-keto-3-deoxy-arabonate compared to the wildtype enzyme. Additionally, biosynthesis was further enhanced through the balancing of carbon flux distribution via modular optimization. The best engineered strain produced 705.1 mg/L BTO from arabinose in shake flask experiments. This work demonstrates a promising microbial platform for BTO production and expands the repertoire of products derived from arabinose, laying the foundation for the comprehensive utilization of lignocellulosic components.

## Methods

### Medium, strains and plasmids

Luria–Bertani (LB) medium was utilized for plasmid propagation, cell inoculation, and protein expression. This medium consists of the following components per liter: 10 g of tryptone, 10 g of NaCl, and 5 g of yeast extract. For biosynthesis of BTO, we employed M9 medium with the following composition per liter: 10 g of glucose, 10 g of arabinose, 5 g of yeast extract, 2 g of NH_4_Cl, 6 g of Na_2_HPO_4_, 3 g of KH_2_PO_4_, 0.5 g of NaCl, 2 g of MOPS, 1 mM of MgSO_4_, and 0.1 mM CaCl_2_. If necessary, the antibiotics ampicillin, kanamycin and chloramphenicol were added to the medium at their respective final concentrations of 100 μg/mL, 50 μg/mL and 34 μg/mL. For protein expression and production of BTO, Isopropyl beta-D-thiogalactopyranoside (IPTG) was added at a concentration of 0.5 mM when the optical density (OD_600_) of the cells reached 0.6. The *E. coli* strain DH5α was employed for plasmid construction, while the *E. coli* strain BW25113 (F') was used for production of BTO. In the process of pathway construction, we utilized the high-copy number plasmid pZE12-luc, the medium-copy number plasmid pCS27, and the low-copy number plasmid pSA74. The list of the strains and plasmids utilized in this study are shown in the Table 1.

**Table 1:** Strains and plasmids used in this study

Strain	Genotype	Source
*E. coli* BW25113/F’	*Δ(araD-araB)567, ΔlacZ4787(::rrnB-3), λ-, rph-1, Δ(rhaD-rhaB)568, hsdR514 [F’ proAB lacIqZDM15Tn10 (TetR)]*	Yale CGSC
*E. coli* Trans5α	*F-φ80d lacZΔM15 Δ(lacZYA-argF) U169 end A1 recA1 hsdR17 (rk -, mk* +*) supE44λ- thi-1 gyrA96 relA1 phoA*	Stratagene
*E. coli* BL21(DE3)	*F*^*−*^*ompT hsdS*_*B*_* (r*_*B*_^*−*^*m*_*B*_^*−*^*) gal dcm (DE3)*	Invitrogen
S01	BW25113/F′ harboring pZE12-araABC and pCS-mdlC-yqhD	This study
S02	BW25113/F′ harboring pZE12-araABC and pCS-aro10-yqhD	This study
S03	BW25113/F′ harboring pZE12-araABC and pCS-kivD-yqhD	This study
S04	BW25113/F′ harboring pZE12-araABC and pCS-kivD(V461I)-yqhD	This study
S05	BW25113/F′ harboring pZE12-araABC and pCS-kivD(S286T)-yqhD	This study
S06	BW25113/F′ harboring pZE12-araABC and pCS-kivD(V461I/S286T)-yqhD	This study
S07	BW25113/F′ harboring pCS-araABC, pZE-kivD(V461I/S286T)-yqhD	This study
S08	BW25113/F′ harboring pCS-araABC-yqhD, pZE-kivD(V461I/S286T)	This study
S09	BW25113/F′ harboring pCS-araABC, pZE-kivD(V461I/S286T) and pSA-yqhD	This study

Plasmid	Description	Source
pZE12-luc	pLlacO1; *luc*; *ColE1 ori*; *Amp*^*R*^	[6]
pCS27	pLlacO1; *p15A ori; Kan*^*R*^	[8]
pSA74	PLlacO1; *pSC101 ori; CmR*	[29]
pETDuet-1	two T7 promoters; two MCS; *pBR322 ori*; *Amp*^*R*^	Novagen
pZE-araABC	pZE12 containing *araA*, *araB* and *araC* from *Burkholderia multivorans*	This study
pCS-mdlC-yqhD	pCS27 containing *mdlC* from *Pseudomonas putida* ATCC12633 and *yqhD* from *E. coli*	This study
pCS-aro10-yqhD	pCS27 containing *aro10* from *Saccharomyces cerevisiae* and *yqhD* from *E. coli*	This study
pCS-kivD-yqhD	pCS27 containing *kivD* from *Lactococcus lactis* and *yqhD* from *E. coli*	This study
pCS-kivD(V451I)-yqhD	pCS27 containing Kivd mutant V451I and *yqhD* from *E. coli*	This study
pCS-kivD(S286T)-yqhD	pCS27 containing Kivd mutant S286T and *yqhD* from *E. coli*	This study
pCS-kivD(V451I/S286T)-yqhD	pCS27 containing Kivd mutant V451I/S286T and *yqhD* from *E. coli*	This study
pCS-araABC	pCS27 containing *araA*, *araB* and *araC* from *Burkholderia multivorans*	This study
pCS-araABC-yqhD	pCS27 containing *araA*, *araB* and *araC* from *Burkholderia multivorans* and *yqhD* from *E. coli*	This study
pZE-kivD(V451I/S286T)	pZE12 containing Kivd mutant V451I/S286T	This study
pZE-kivD(V451I/S286T)-yqhD	pZE12 containing Kivd mutant V451I/S286T and *yqhD* from *E. coli*	This study
pSA-yqhD	pSA74 containing *yqhD* from *E. coli*	This study

### DNA manipulation

The genes *araA*, *araB* and *araC* from *B. multivorans* were codon optimized and synthesized by Sangon Biotech (Shanghai, China). They were cloned into the pZE12-luc vector using the *K*pnI, *P*stI, *B*amHI and *X*baI sites to generate the plasmid pZE-araABC. The genes *mdlC*, *aro10*, *kivD* and *yqhD* were amplified from the genome of *P. putida* ATCC12633, *S. cerevisiae*, *L. lactis* and *E. coli*, respectively. These three genes were separately cloned with *yqhD* into the pCS27 vector using *K*pnI, *S*phI and *S*alI sites to generate plasmids pCS-mdlC-yqhD, pCS-aro10-yqhD and pCS-kivD-yqhD, respectively. The KivD mutants were created by overlap PCR using appropriate primers. The generated PCR products were purified inserted into the plasmid pCS-kivD-yqhD to replace the wildtype KivD, yielding plasmids pCS-KivD(V461I)-yqhD, pCS-KivD(S286T)-yqhD and pCS-KivD(V461I/S286T)-yqhD. All mutants were subjected to sequencing to confirm the accuracy of the mutations. To purify KivD and its mutants, the wildtype KivD and its mutants were cloned into the pETDuet-1 vector between *B*amHI and *K*pnI sites to yield plasmid pET-kivD, pET-kivD(V461I), pET-kivD(S286T) and pET-kivD(V461I/S286T). The plasmid pCS-araABC was created by inserting *araA*, *araB* and *araC* into the pCS27 backbone using the *K*pnI, *S*phI, *P*stI and *B*amHI sites. The plasmid pCS-araABC-yqhD was constructed by inserting the expression cassette PLlacO1-yqhD into the plasmid pCS-araABC between *S*acI and *S*peI sites. The plasmid pZE-kivD(V461I/S286T) was created by inserting kivD(V461I/S286T) into the plasmid pZE12 vector between *K*pnI and *X*baI sites. The plasmid pZE-kivD(V461I/S286T)-yqhD was constructed by inserting the expression cassette PLlacO1-yqhD into the plasmid pZE-kivD(V461I/S286T) between *S*acI and *S*peI sites. The plasmid pSA-yqhD was created by inserting the gene *yqhD* into the pSA74 vector between *K*pnI and *B*amHI sites.

### In vitro enzyme assays

The plasmids pET-kivD, pET-kivD(V461I), pET-kivD(S286T) and pET-kivD(V461I/S286T) were individually introduced into *E. coli* BL21 Star (DE3) for protein purification. The transformants were initially cultured in 4 mL LB medium supplemented with 100 μg/mL ampicillin at 37 ℃ overnight. Then, 1 mL of these cultures was transferred into 50 mL of fresh LB medium containing 100 μg/mL ampicillin at 37 ℃ until the OD_600_ value reached 0.6. At this stage, 0.5 mM IPTG was added into the culture and the cells were cultivated at 30 ℃ for overnight. Subsequently, the cells were harvested then lysed by an ultrasonic apparatus (JY92-IIN, SCIENTZ). The recombinant protein was purified using the His-Spin protein miniprep kit from ZYMO RESEARCH. The concentration of the purified protein was determined using a BCA kit from Pierce Chemicals. The enzyme activities were measured through coupled enzyme assays with AraC. The enzyme assays were performed by adding 500 nM purified wild type or mutated KivD proteins into the reaction mixtures which contain excess AraC (2 mM),

100 mM MgCl_2_, 1 mM TPP. The final volume was adjusted to 1 mL with Tris–HCl buffer (100 mM, pH = 7.5). A gradient concentration of substrate arabinate ranging from 0 to 2000 μM was employed. The reaction system was maintained at 30 ℃ for 1 h. Then 2% TFA was added into the mixture to stop the reaction. 300 μL of semicarbazide hydrochloride (0.1 mM) was added and reacted at 30 ℃ for 30 min. The absorbance of the semicarbazone was assessed at a wavelength of 250 nm. The decarboxylase activities of KivD and its mutants were determined by observing the reduction in absorbance at 250 nm, which resulted from the consumption of 2-keto-3-deoxy-arabinate. Apparent kinetic parameters were determined by non-linear regression analysis using the Michaelis–Menten equation with OriginPro8.5TM. Each experiment was conducted at least in duplicates.

### Shake flask experiments

For shake flask experiments, the M9 medium containing 10 g/L glucose and 10 g/L arabinose was used. The transformants of the *E. coli* strain BW25113 (F’) were initially inoculated in 4 mL LB medium and incubated overnight at 37 ℃ with 220 rpm. Then, 1 mL of this culture served as the pre-inoculum and transferred into 50 mL of M9 medium. If needed, the final concentration of 100 μg/mL ampicillin, 50 μg/mL kanamycin and 34 μg/mL chloramphenicol was added into the cultures. The cultures were incubated at 37 ℃ until the OD_600_ reached 0.6. Following this, IPTG was introduced into the cultures to a final concentration of 0.25 mM. The cells were then transferred to shakers operating at 30 ℃ with 220 rpm. Samples were collected periodically during the experiments. The optical density at 600 nm (OD_600_) of the cultures was measured, and the concentrations of both the products and intermediates were analyzed using high-performance liquid chromatography (HPLC).

### Analytical procedures

The OD_600_ values were measured by UV spectrophotometer (Thermo Fisher Scientific, MA, USA). After collecting the samples, they were centrifuged at 13,000 rpm for 10 min. The resulting supernatant was then filtered through a 0.22 μm polyethersulfone film and subsequently analyzed and quantified using an HPLC system. The HPLC was equipped with an organic acid analysis column (Amine HPX-87H ion exclusion column, 300 mm × 7.8 mm) and refractive index detector. The eluent phase employed in the analysis was 5 mM H_2_SO_4_. The column temperature was set at 40 ℃ and the flow rate was maintained at 0.5 mL/min.

## Results

### Designing an artificial biosynthetic pathway for BTO production from arabinose

**Fig. 1:**
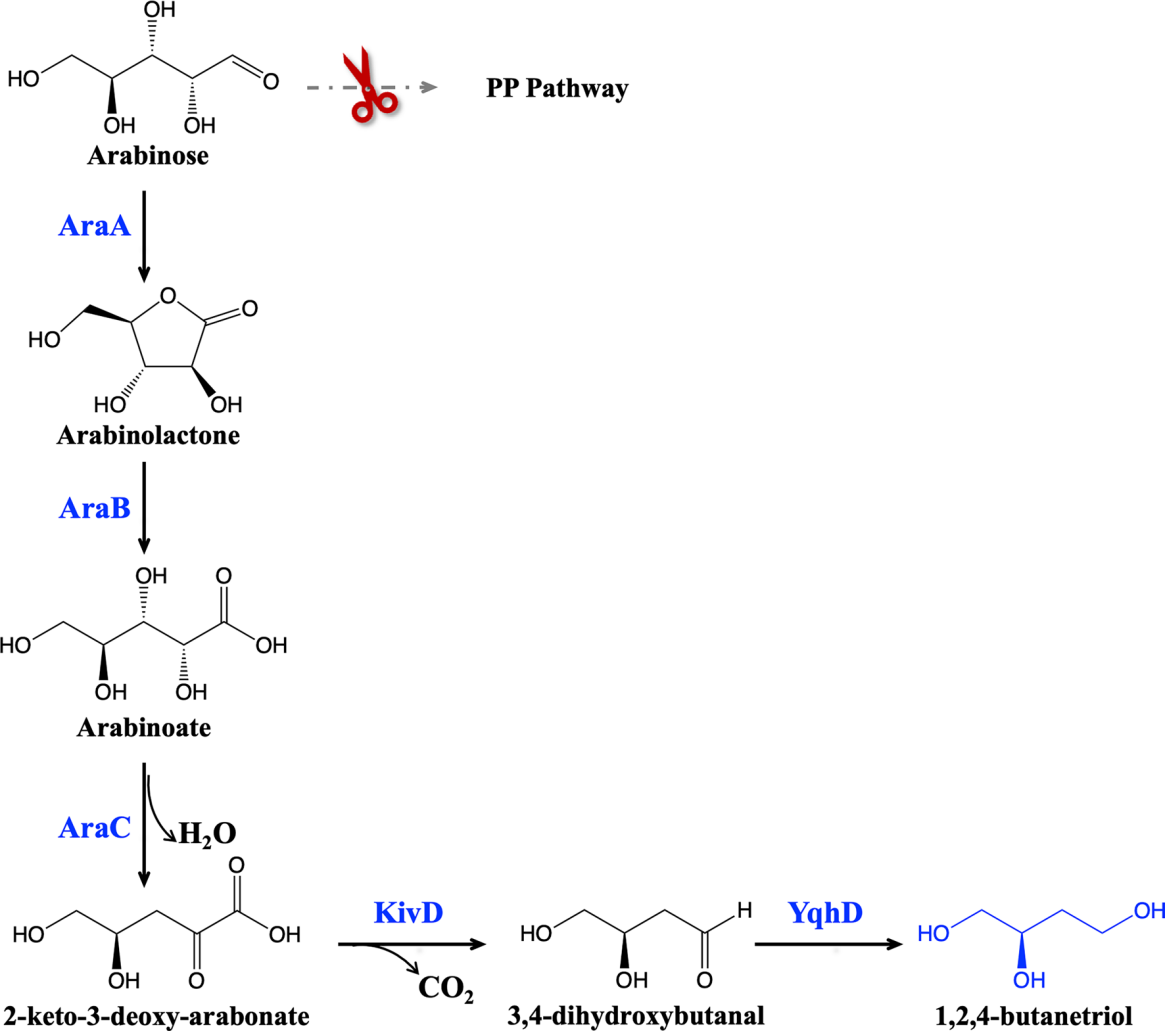
The artificial biosynthetic pathway for the production of BTO from arabinose in *E. coli*. *AraA* arabinose dehydrogenase, *AraB* arabinolactonase, *AraC* arabonate dehydratase, *KivD* keto acid decarboxylase, *YqhD* alcohol dehydrogenase, *PP* pathway, pentose phosphate pathway. Solid line indicates a single step; dotted line indicates multiple steps

In this current study, we present an innovative biosynthetic pathway for the synthesis of BTO from arabinose (Fig. 1). The process involves several enzymatic steps. Initially, arabinose undergoes conversion into arabinolactone, facilitated by arabinose dehydrogenase. Subsequently, arabinolactone is transformed into arabonate with the assistance of arabinolactonase. Following this, arabonate dehydratase plays a key role in converting arabonate into 2-keto-3-deoxy-arabonate, which is further metabolized into 3,4-dihydroxybutanal through the catalysis of keto acid decarboxylase. Lastly, the 3,4-dihydroxybutanal is converted into BTO via alcohol dehydrogenase. Notably, it has been demonstrated that arabinose dehydrogenase (AraA), arabinolactonase (AraB), and arabonate dehydratase (AraC) from *Burkholderia multivorans* efficiently catalyze the conversion of arabinose to 2-keto-3-deoxy-arabonate in the initial three steps [4]. While keto acid decarboxylases encompass a diverse range of enzymes capable of decarboxylating various keto acids [21], an efficient keto acid decarboxylase for 2-keto-3-deoxy-arabonate has not been reported to date. In *E. coli*, a wide range of native alcohol dehydrogenases are available for converting aldehydes to alcohols [22]. It is worth noting that our prior research has revealed that the *E. coli* native aldehyde reductase, YqhD, has the capability to reduce 3,4-dihydroxybutanal to yield BTO [6].

### Screening of a ketoacids decarboxylase for BTO production

To assemble the entire BTO biosynthetic pathway, the identification of an efficient 2-keto-3-deoxy-arabonate decarboxylase is crucial. Several candidate enzymes exhibit potential due to their broad substrate specificity. Specifically, the benzoylformate decarboxylase MdlC from *Pseudomonas putida* ATCC12633 is highly specific for benzoylformate, while this enzyme also demonstrates the capability to accept a range of large aromatic and small aliphatic 2-keto acids [23]. The phenylpyruvate decarboxylase Aro10 from *Saccharomyces cerevisiae* has a wide range of substrate specificity towards various keto acids [24]. In addition, we previously reported that a branched-chain keto acids decarboxylase KivD from *Lactococcus lactis* displayed significant activity towards 2-keto-3-deoxy-xylonate [8]. The adaptability of these three enzymes in accommodating a variety of keto acids suggests that they may effectively function as 2-keto-3-deoxy-arabonate decarboxylases. However, further research and experimentation are necessary to confirm their suitability for this specific role in the BTO biosynthetic pathway.

**Fig. 2:**
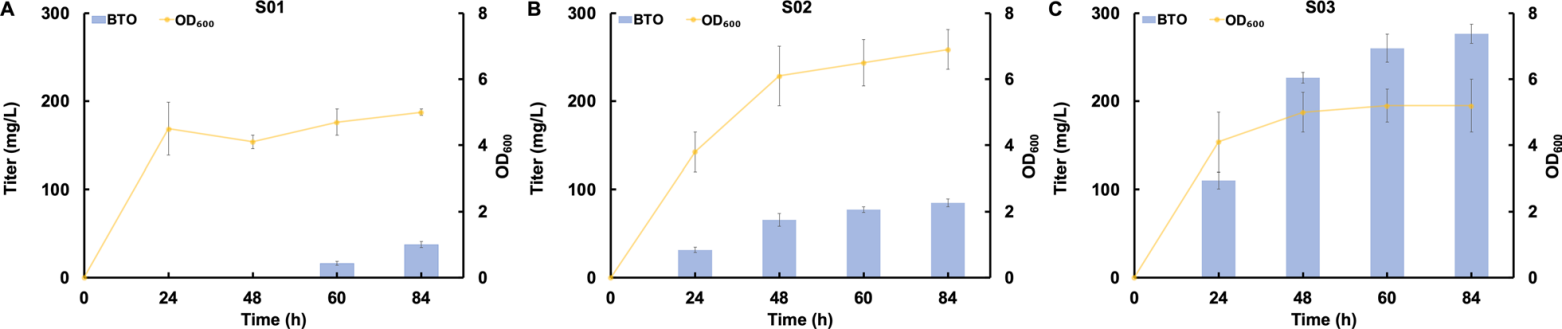
Screening of a superior keto acid decarboxylase. **A** Strain S01 harboring pZE12-araABC and pCS-mdlC-yqhD, **B** Strain S02 harboring pZE12-araABC and pCS-aro10-yqhD, **C** Strain S03 harboring pZE12-araABC and pCS-kivD-yqhD. All data points are reported as mean ± s.d. from three independent experiments

To assess their catalytic activity, we introduced these three enzymes into the BTO biosynthetic pathway. The genes *araA*, *araB*, and *araC* were cloned into the high-copy number plasmid pZE12-luc, resulting in the plasmid pZE-araABC. Simultaneously, the genes *mdlC*, *aro10*, and *kivD* were individually cloned, along with *yqhD*, into the medium-copy number plasmid pCS27, leading to the plasmids pCS-mdlC-yqhD, pCS-aro10-yqhD and pCS-kivD-yqhD, respectively. The *E. coli* BW25113 (F') strain, devoid of arabinose isomerase, served as the host strain to prevent the assimilation of arabinose into the pentose phosphate pathway. The co-transfer of plasmid pZE-araABC with each of these three plasmids into the *E. coli* BW25113 (F’) strain resulted in the strains S01, S02, and S03. As shown in the Fig. 2, the BTO-producing strain S01 and S02 expressing *mdlC* and *aro10* synthesized only 37.5 mg/L and 84.9 mg/L BTO from 10 g/L arabinose, respectively (Fig. 2A and B). While, the strain S03 harboring *kivD* produced the highest amount of BTO, reached 276.7 mg/L (Fig. 2C). These results indicated that KivD is the most efficient keto acid decarboxylase for the conversion of 2-keto-3-deoxy-arabonate. However, we also observed that more than 9 g/L arabinose accumulated in the medium, suggesting that the poor activity of KivD acts as a bottleneck in the BTO biosynthetic pathway.

### Designing KivD mutants to improve the activity towards 2-keto-3-deoxy-arabonate

The discovery of KivD enabled the biosynthesis of BTO from arabinose, however, its low catalytic efficiency rendered it unsuitable as a biocatalyst for the decarboxylation step in the biosynthetic pathway. To enhance BTO production, we investigated the impact of protein engineering on improving KivD activity towards 2-keto-3-deoxy-arabonate. KivD is a thiamin diphosphate (TPP) dependent decarboxylase that natively convert 3-methyl-2-ketobutanoic acid to 3-methylpropanal and CO_2_ [25]. Previous research has shown that KivD displays significantly higher activity with branched-chain substrates compared to single-chain substrate [26]. Given that 2-keto-3-deoxy-arabonate lacks branch-chain and is a narrow substrate than 3-methyl-2-ketobutanoic acid, shrinking the binding pocket size is very promising for improving its activity towards the smaller substrate 2-keto-3-deoxy-arabonate.

**Fig. 3:**
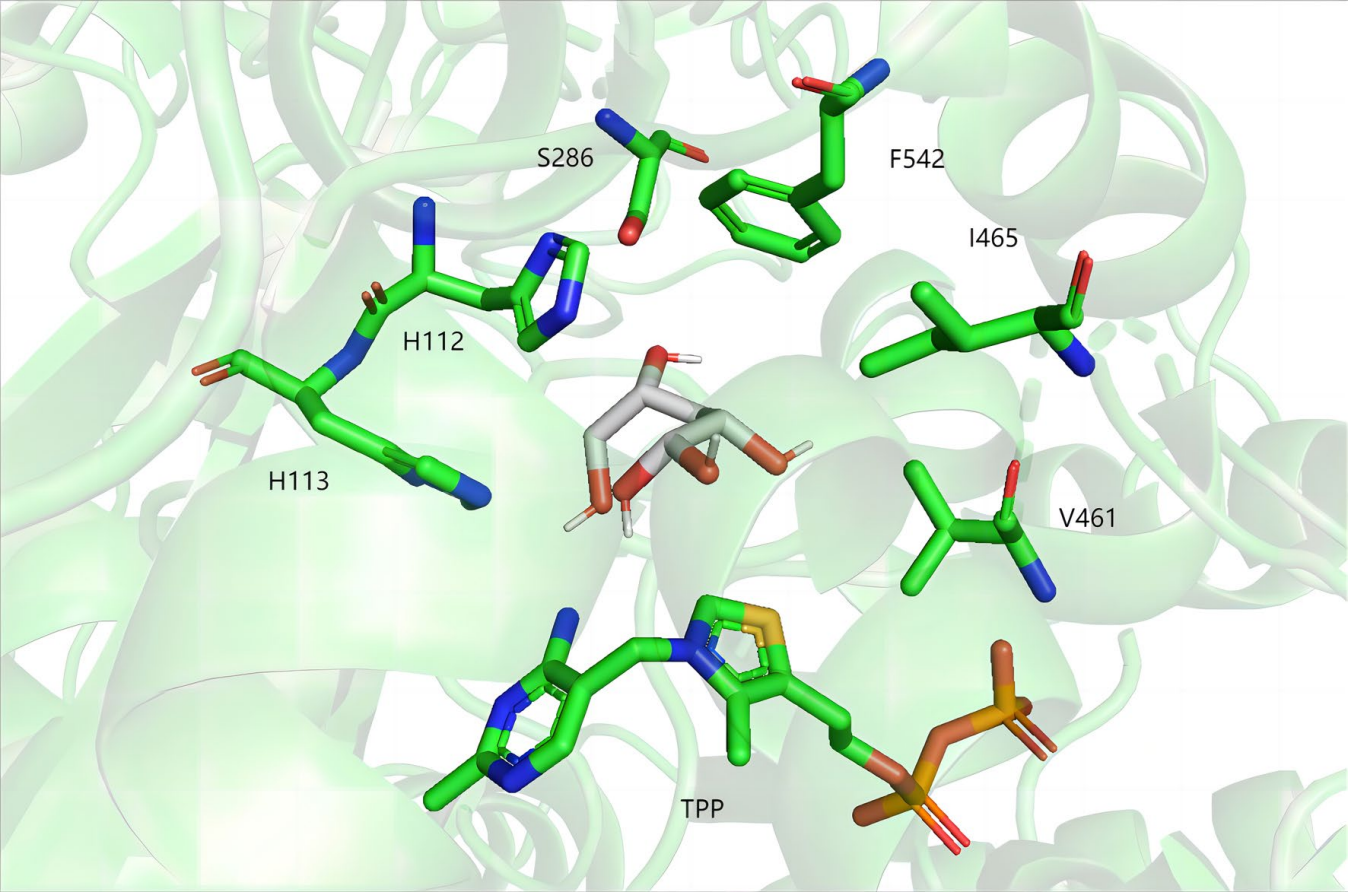
Representation of the catalytic pocket of KivD generated by molecular docking models. His112 and His113 are the two active-site residues of KivD. The binding site of KivD is delineated by amino acid residues V461, I465, S286 and F542 in conjunction with the cofactor thiamin diphosphate (TPP)

To examine our hypothesis, we conducted a docking study utilizing the target substrate 2-keto-3-deoxy-arabonate. As shown in the Fig. 3, the active-site residues of Kivd include two catalytic histidines (His112 and His113). Additionally, the binding site of KivD is defined by amino acid residues V461, I465, S286 and F542 in conjunction with the cofactor TPP (Fig. 3). We focus on the substitution of the smaller amino acid residues V461 and S286 with larger counterparts. Previous research suggested that replacing V461 with isoleucine and S286 with threonine could effectively reduce the binding pocket size and redirect KivD preference towards the smaller substrate, 2-ketoisovalerate, rather than the larger substrate, 2-ketoisocaproate [27]. However, whether these substitutions can also enhance KivD performance towards 2-keto-3-deoxy-arabonate remains unknown.

### Constructing and charactering KivD mutants

**Fig. 4:**
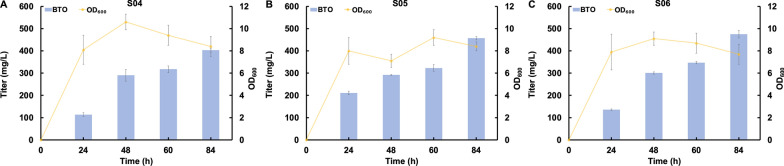
Biosynthesis of BTO from arabinose using KivD mutants. **A** Strain S04 harboring pZE12-araABC and pCS-kivD(V461I)-yqhD, **B** Strain S05 harboring pZE12-araABC and pCS-KivD(S286T)-yqhD, **C** Strain S06 harboring pZE12-araABC and pCS-KivD(V461I/S286T)-yqhD. All data points are reported as mean ± s.d. from three independent experiments

To validate the rationale and hypotheses outlined in the aforementioned design, the single mutation enzymes V461I and S286T were created and tested by shake flask experiments. The plasmids pCS-kivD(V461I)-yqhD and pCS-kivD(S286T)-yqhD were constructed and separately co-transferred with the plasmid pZE-araABC, generating the strains S04 and S05, respectively. As depicted in the Fig. 4A and B, the V461I and S286T variants produced 403.4 mg/L and 457.0 mg/L BTO from arabinose, respectively, representing a 45.8% and 65.2 increase compared to the wildtype KivD. Given the better performance of V461I and S286T, we proceeded to create double mutation enzyme by combining the engineered variants, resulting in the KivD S286T/V461I mutant. The plasmid pCS-kivD(S286T/V461I)-yqhD was co-transferred with the plasmid pZE-araABC to yield the strain S06. As expected, the BTO titer was further enhanced to 475.1 mg/L (Fig. 4C), which is 71.7% higher than the wildtype KivD.

We subsequently proceeded to assess the in vitro catalytic efficiency of the KivD mutants through coupled enzyme assays employing purified protein. As shown in the Table 2, both V461I and S286T mutants exhibited increased substrate binding affinity (*K*_m_) compared to the wildtype KivD, from 0.940 mM to 0.572 mM and 0.423 mM, respectively. Notably, the catalytic efficiency of the S286T/V461I mutant towards 2-keto-3-deoxy-arabonate (*k*_cat_/*K*_m_ = 1.189 mM^−1^ s^−1^) was nearly threefold higher as compared to the wildtype KivD (*k*_cat_/*K*_m_ = 0.421 mM^−1^ s^−1^). These enzyme characterization data were in accordance with the results of the shake flask experiments.

**Table 2:** Kinetic parameters of Kivd and its mutants toward 2-keto-3-deoxy-arabonate

Enzyme	Substrate	*K*_*m*_ (mM)	*k*_*cat*_ (s^−1^)	*k*_*cat*_/*K*_*m*_ (mM^−1^ s^−1^)
KivD	2-Keto-3-deoxy-arabonate	0.940 ± 0.127	0.396 ± 0.023	0.421
KivD V461I	2-Keto-3-deoxy-arabonate	0.572 ± 0.133	0.462 ± 0.036	0.807
KivD S286T	2-Keto-3-deoxy-arabonate	0.423 ± 0.070	0.458 ± 0.021	1.084
KivD V461I/S286T	2-Keto-3-deoxy-arabonate	0.278 ± 0.045	0.332 ± 0.012	1.189

Enzyme activity was determined using a coupled enzyme assay. Data are presented as mean ± s.d. (*n* = 3)

### Modular optimization to further improve BTO production

**Fig. 5:**
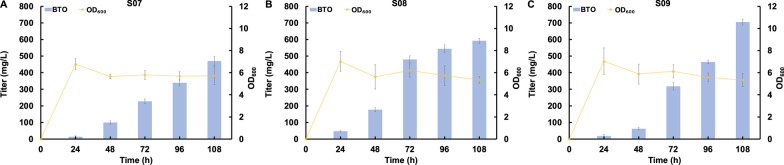
Improvement of BTO production by modular optimization. **A** Strain S07 harboring pCS-araABC and pZE-KivD(V461I/S286T)-yqhD, **B** Strain S08 harboring pCS-araABC-yqhD and pZE-KivD(V461I/S286T), **C** Strain S09 harboring pCS-araABC, pZE-KivD(V461I/S286T) and pSA-yqhD. All data points are reported as mean ± s.d. from three independent experiments

To further improve BTO production, we conducted modular optimization aimed at balancing the carbon flux through the BTO biosynthetic pathway. Given the higher bioconversion efficiency of arabinose to arabinate catalyzed by AraABC in comparison to the decarboxylation reaction catalyzed by KivD, we hypothesized that down-regulating the expression level of *araABC* and up-regulating the expression level of KivD could optimize the carbon flux through the whole pathway. Consequently, we placed the genes *araA*, *araB* and *araC* on a medium-copy number plasmid to generate the plasmid pCS-araABC. Simultaneously, we introduced the KivD mutant S286T/V461I on a high-copy number plasmid, designated as pZE-kivD(S286T/V461I). We also fine-tuned the expression level of the alcohol dehydrogenase YqhD by utilizing high, medium, or low-copy number plasmids. As the results shown in the Fig. 5, the highest BTO titer was obtained by the strain S09 carrying the plasmids pCS-araABC, pZE-kivD(S286T/V461I) and pAS-yqhD (Fig. 5A). While, further enhancing the expression level of *yqhD* by placing it on a medium-copy number plasmid (strain S08) or high-copy number plasmid (strain07) led to decreased BTO titers to 592.0 and 469.6 mg/L, respectively (Fig. 5B and C), probably duo to the increased metabolic burden. In summary, through modular optimization, the best-performing strain S09 synthesized 705.1 mg/L of BTO from arabinose, marking a remarkable 48.4% improvement when compared to the control strain S06.

## Discussion

Lignocellulose feedstock represents a large amount and sustainable raw materials for the production of a diverse range of chemicals. Arabinose is the third most abundant sugar in lignocellulose follows glucose and xylose, with its content in lignocellulose ranging from 1 to 18%. However, arabinose has received relatively little attention in terms of its conversion into value-added compounds. In this study, we successfully achieved microbial production of the high value molecule BTO from arabinose, which is of great significance in the pursuit of fully utilizing lignocellulosic components. To construct BTO biosynthetic pathway, we harnessed the inherent arabinose metabolic pathway within *B. multivorans.* This allowed us to convert arabinose into 2-keto-3-deoxy-arabonate. We then screened ketoacids decarboxylase to facilitate the production of BTO. This methodology can be readily extended to synthesize BTO from other sugars, such as fucose, rhamnose and galacturonate [28].

In this work, we identified the keto acid decarboxylase KivD as the most efficient enzyme for decarboxylation of 2-keto-3-deoxy-arabonate. To further improve KivD activity, we employed protein engineering through a rational design approach. This involved reducing the size of the binding pocket to accommodate a smaller substrate. The application of this approach led to an improvement in KivD's activity, with the engineered enzyme exhibiting nearly a threefold increase in efficiency compared to the native KivD when catalyzing 2-keto-3-deoxy-arabonate. It is worth noting that the KivD demonstrates much higher catalytic efficiency when acting on 2-keto-3-deoxy-xylonate compared to its performance with 2-keto-3-deoxy-arabonate. For example, we previously reported that an engineered *E. coli* strain expressing KivD can produce 1.5 g/L BTO when using xylose as the carbon source [6]. The structural difference between 2-keto-3-deoxy-xylonate and 2-keto-3-deoxy-arabonate is the configuration of the hydroxyl group at the C4 site. Therefore, we believe that analysis and mutation of the amino acids residues that interact with the C4 hydroxyl group hold promise for further improving Kivd activity towards 2-keto-3-deoxy-arabonate. In addition, it has been reported that having a sufficient supply of NADH is beneficial for BTO production when utilizing xylose as the carbon source [20]. This strategy can also be applied to increase BTO titer in the arabinose-based biosynthetic pathway.

## Conclusions

In summary, our study involved the development of an artificial biosynthetic pathway for producing BTO from arabinose in *E. coli*. Through a combination of screening and protein engineering of the keto acid decarboxylase KivD, we greatly enhanced the production efficiency. Additional improvements were achieved through modular optimization of carbon flux distribution. The best-performing engineered strain produced 705.1 mg/L of BTO from arabinose in shake flask experiments. This research extends the spectrum of products that can be derived from arabinose, making a contribution to the comprehensive utilization of lignocellulosic components.

## Data Availability

All data generated or analyzed during this study are included in this published article.
